# The Impact of Retirement Resources on U.S. Older Female Workers’ Retirement Timing: Theory of Planned Behavior Model

**DOI:** 10.1093/geroni/igab046.3738

**Published:** 2021-12-17

**Authors:** Hansol Kim, David Ekerdt, Tamara Baker, Amber Watts, Tracey LaPierre, Yan Bing Zhang

**Affiliations:** 1 University of Pittsburgh, Pittsburgh, Pennsylvania, United States; 2 University of Kansas, Lawrence, Kansas, United States; 3 University of North Carolina, Chapel Hill School of Medicine, University of North Carolina at Chapel Hill (School of Medicine), North Carolina, United States

## Abstract

For older workers, having a retirement plan is important for a successful transition. Social awareness of the problems encountered by older women during retirement remains low. Women have limited retirement resources due to their unequal work experience, and older women with access to fewer retirement resources often postpone their retirement. This research examined how the timing of older women’s retirement was influenced by their retirement resources as well as their marital status. The study used 2014 HRS and RAND data, and collected sample of women aged 50-62 years old who worked either full or part time (n=3,593). Respondents were female (56%), white (63%), married (70%), and working full time (82%). Guided by the theory of planned behavior (TPB), multiple regression analysis examined gender differences in predicting older adults' retirement timing. TPB included three sub factors: attitudes toward retirement, subjective norm, and perceived behavioral control. Logistic regression analyzed the effects of respondents’ expectations of retirement (i.e., with vs without expected timing). The findings indicated that the TPB model works similarly for men and women but there is a difference according to marital status. Unmarried women are less likely to have accumulated financial resources and more likely to anticipate a later retirement (1.4 years) than married women and are also less likely to set an expected timing for retirement (p<05). Such a robust research agenda would provide key information for government agencies and policymakers and contribute to the development of retirement planning models or retirement education programs for older women.

